# Physical Activity and Sedentary Behavior Patterns Among Korean Cancer Survivors: A Cross-Sectional Analysis (2017–2021)

**DOI:** 10.3390/cancers17142270

**Published:** 2025-07-08

**Authors:** Jiin Ryu, Jihee Min

**Affiliations:** 1 Department of Applied Physiology and Kinesiology, University of Florida, Gainesville, FL 32611, USA; ryu.j@ufl.edu; 2 National Cancer Survivorship Center, National Cancer Control Institute, National Cancer Center, Goyang-si 10408, Republic of Korea

**Keywords:** cancer survivors, domain-specific physical activity, sedentary behavior, Korean adults

## Abstract

**Background/Objectives**: This study examined differences in physical activity (PA) and sedentary behavior among Korean cancer survivors compared to cancer-free individuals from 2017 to 2021 and identified differences based on their cancer treatment status. **Methods**: We used data for 28,528 adults (1585 cancer survivors and 26,943 cancer-free individuals) from the 2017–2021 Korea National Health and Nutrition Examination Survey. PA and sedentary behavior were assessed using the Korean version of the Global Physical Activity Questionnaire. We examined adherence to aerobic and resistance training guidelines, domain-specific activity, and sedentary time. We also analyzed the descriptive statistics and conducted chi-square tests, the Mann–Whitney U test, and the non-parametric Kruskal–Wallis test, stratified by demographic and clinical subgroups. **Results**: Compared with cancer-free individuals, cancer survivors showed significantly lower adherence to aerobic PA guidelines in 2017 (*p* < 0.01), 2018 (*p* < 0.05), and 2021 (*p* < 0.01). Transportation-related activity accounted for the highest share of the total PA (mean: 59.1%). Sedentary time increased among survivors from 8.1 h/day (2017) to 9.0 h/day (2021). In 2021, resistance training adherence was higher among survivors (24.7%) than among non-cancer individuals (22.9%). In the subgroup analyses, male sex, younger age, recent diagnosis, higher income, and living with a spouse were associated with higher adherence. **Conclusions**: The findings underscore a clear need to address both PA and sedentary behavior in cancer survivors, irrespective of treatment phase.

## Simple Summary

Physical activity is closely linked to cancer prevention, recovery, and managing treatment-related side effects throughout the cancer care continuum. However, most cancer survivors face a struggle with maintaining an active lifestyle and often spend long periods marked with sedentary behaviors compared to individuals without cancer. This study examined the annual variations in physical activity and sedentary behaviors among Korean cancer survivors between 2017 and 2021, comparing them to those without cancer. We found cancer survivors were less likely to meet the recommended levels of aerobic activity, and much of their movement was related to transportation rather than leisure activity. These findings suggest that the need for a broader strategy that not only promotes physical activity but also reduces prolonged sitting. Supporting behavioral change through social and financial support, along with culturally appropriate programs, may help cancer survivors develop healthier and more sustainable movement habits.

## 1. Introduction

Globally, cancer incidence continues to rise, with approximately 20 million new cases reported in 2022 [1,2], alongside improved survival rates attributable to advancements in early detection and therapeutic interventions [3]. In South Korea, although cancer has long been the leading cause of death, advances in detection and treatment have now increased the five-year relative survival rate to 72.9% as of 2022, resulting in cancer survivors accounting for about 5% of the total South Korean population [4]. This rapidly expanding survivor population faces unique long-term health challenges [5]. Consequently, there is a burgeoning public health imperative to address the chronic disease risks and management needs of this population, focusing on strategies to optimize their overall well-being and long-term prognosis [6,7].

Physical activity (PA) is increasingly recognized as a cornerstone of both cancer prevention and survivorship care [8,9]. A robust evidence base shows that regular PA can reduce the risk of cancer recurrence [10], mitigate treatment-related adverse effects, and enhance the overall survival of cancer survivors [11,12,13]. Furthermore, PA has been associated with a decreased risk of developing concomitant chronic conditions, such as cardiovascular disease [14], diabetes mellitus [15], and osteoporosis [16], which are frequently observed in cancer survivors. Conversely, prolonged sedentary behavior, particularly extended sitting time, has emerged as an independent risk factor for cancer incidence and cancer-specific mortality, regardless of PA levels [17]. For example, higher levels of sedentary behavior were associated with 54% and 66% increases in the risks of colorectal and endometrial cancer, respectively [18], while prolonged sitting has been linked to a 12–22% higher risk of cancer mortality among the least active individuals [19]. Thus, a dual focus on increasing PA and reducing sedentary time is essential for comprehensive cancer care.

Although previous studies have evaluated PA levels at single time points or assessed the effects of specific interventions [20,21], cross-sectional time-series analyses of both PA and sedentary behavior in cancer survivors are scarce. Furthermore, despite evidence that work-related PA may elevate mortality risk while leisure-time PA may confer protective benefits [22,23,24], research characterizing domain-specific PA patterns—related to occupation, transportation, and leisure-time—remains limited.

Therefore, this study conducted a comparative analysis of adherence to PA guidelines and sedentary behavior patterns among Korean cancer survivors and cancer-free individuals, using independent annual samples from the Korea National Health and Nutrition Examination Survey (KNHANES) for 2017–2021. Additionally, we sought to characterize the factors influencing PA patterns by identifying characteristics associated with domain-specific PA engagement among cancer survivors.

## 2. Methods

### 2.1. Study Design and Data Source

This cross-sectional study utilized data from KNHANES for 2017–2021. KNHANES is a nationally representative, population-based survey administered annually by the Korea Disease Control and Prevention Agency to monitor the health and nutritional status of the South Korean population. The survey employs a stratified, multistage probability sampling design. All data were collected through standardized in-person interviews, physical examinations, and self-reported questionnaires.

### 2.2. Study Population

This study analyzed data from the 2017–2021 KNHANES. From an initial dataset of 38,678 individuals, we excluded participants under 19 years of age, those with incomplete physical activity data, and those with missing data on cancer-related questions (n = 10,150). The final analytic sample comprised 28,528 participants. (Supplementary Figure S1). Participants were categorized into two groups based on their cancer status: cancer survivors (n = 1585; 5.6%), defined as individuals who reported a previous physician diagnosed cancer, and cancer-free individuals (n = 26,943; 94.4%), who reported never having received a cancer diagnosis. After applying sample weights, the analytic sample represented an estimated 1.8 million Korean adults, comprising about 102,608 cancer survivors and 1,729,680 cancer-free individuals. Demographic and clinical characteristics are presented in Table 1.

### 2.3. Definition of Cancer Status

Cancer survivors were identified based on affirmative responses to at least one of the following survey questions: (1) “Have you ever been diagnosed with cancer by a physician?” (2) “Are you currently living with cancer?” (3) “Are you currently undergoing treatment for cancer?” Those who gave affirmative responses to question (3) were considered as currently receiving cancer treatment.

### 2.4. PA and Sedentary Behavior Assessment

PA and sedentary behavior were assessed using the Korean version of the Global Physical Activity Questionnaire (GPAQ), a self-administered questionnaire developed by the World Health Organization. The GPAQ includes 16 items that assess moderate-to-vigorous PA (MVPA) across three domains: occupational, transport-related, and leisure-time activities. For each domain, participants reported the PA’s frequency (days per week) and duration (minutes per day). Total weekly PA was calculated by summing up the minutes per week spent in each domain, with vigorous-intensity activities weighted by a factor of two according to WHO recommendations [25].

Sedentary behavior was assessed using a single item, asking “How much time do you usually spend sitting or reclining on a typical day?” Participants reported their usual average daily sedentary time in hours and minutes. The validity and reliability of the Korean version of the GPAQ have been previously evaluated and found acceptable [26].

### 2.5. Anthropometric and Sociodemographic Parameters

Anthropometry parameters, including weight, height, and waist circumference (WC), were measured following standardized protocols [27]. Body-mass index (BMI) was calculated as weight (kg) divided by height squared (m^2^). Education levels were categorized as follows: less than middle school, high school graduate, and college graduate or higher. Marital status was classified as married or living together, others (including separated, divorced, or widowed), or never married. Household income was categorized into quintiles (low, middle-low, middle, middle-high, or high) based on the equivalized monthly household income.

### 2.6. Statistical Analysis

Participants’ characteristics were summarized using descriptive statistics. Group comparisons between cancer survivors and cancer-free individuals were conducted using independent *t*-tests for continuous variables and chi-square (χ^2^) tests for categorical variables. Due to the non-normal distribution of PA data, median and interquartile range (IQR) are presented in the main analysis, while means and standard deviations (SDs) are provided in Supplementary Materials. 

Moreover, group differences in adherence to aerobic and resistance training guidelines were analyzed at each time point using chi-square (χ^2^) tests. Adherence to PA guidelines was defined as follows: For aerobic activity, meeting the required guideline for at least 150 min per week of moderate-intensity activity or 75 min per week of vigorous-intensity activity; for resistance training, engaging in muscle-strengthening activities at least twice per week [28].

Subgroup analyses were conducted among cancer survivors to examine differences in PA guideline adherence and sedentary behavior based on demographic factors (age, sex, obese status, income level, education level, marital status, and residential type) and clinical characteristics (current cancer treatment status and time since cancer diagnosis). PA guideline adherence by subgroup was analyzed using the chi-square (χ^2^) test.

Descriptive and comparative approaches were used to analyze domain-specific PA, including work-related, transportation-related, and leisure-time activities. Between group comparisons were performed using Mann–Whitney U tests for cancer treatment status and Kruskal–Wallis tests for times since cancer diagnosis, with Bonferroni correction applied to post hoc tests. Cancer type stratification employed the chi-square (χ^2^) test, while patterns of average daily sedentary time across years were evaluated using the Kruskal–Wallis methodology.

All analyses were performed using SPSS software for Windows (version 28.0; IBM Corp., Armonk, NY, USA). All statistical tests were two-sided, and *p* < 0.05 was considered statistically significant.

## 3. Results

### 3.1. Demographic and Clinical Characteristics

Table 1 summarizes the characteristics of the 28,528 participants in this study. The mean age of cancer survivors was 63.0 ± 12.5 years and that of non-cancer individuals was 51.2 ± 17.0 years. The proportion of male participants was lower among cancer survivors (38.4%) compared to cancer-free individuals (44.5%). Cancer survivors were more likely to have lower education levels, be married or living with a partner, and have higher household incomes compared to cancer-free individuals.

Thyroid cancer was the most prevalent (21.2%) cancer type, followed by gastric (14.8%), breast (14.6%), and colorectal (12.0%) cancer. Among cancer survivors, 27.3% were undergoing active cancer treatment, and 36.2% reported a cancer diagnosis within the past five years (Table 1).

### 3.2. PA and Sedentary Behavior (2017–2021)

**Figure 1:**
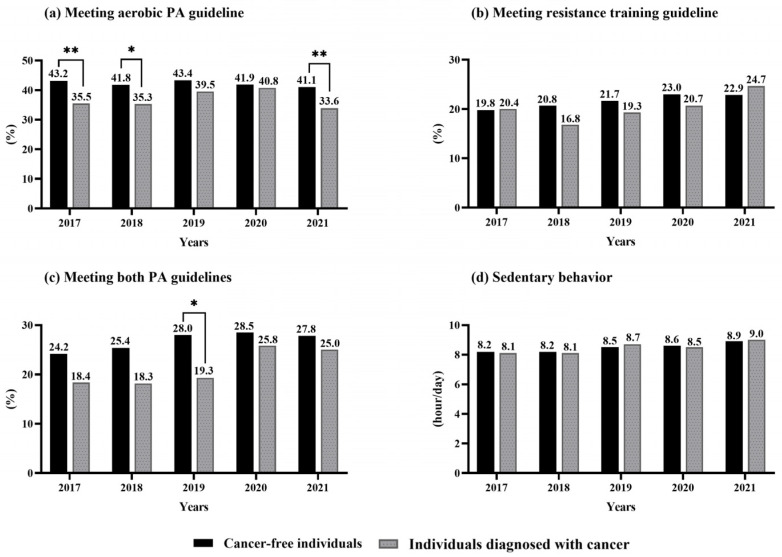
Physical activity (PA) guideline adherence and sedentary behavior among individuals diagnosed with cancer (black) and cancer-free individuals (gray) from 2017 to 2021: (**a**) adherence to the aerobic PA guidelines, (**b**) adherence to the resistance training guidelines, (**c**) adherence to both PA guidelines, and (**d**) sedentary behavior (hours per day). * Indicates significant between-group differences at specific time points. * *p* < 0.05, ** *p* < 0.01. Results are based on the Chi-squared test.

Figure 1 presents cancer survivors’ and non-cancer individuals’ adherence to PA guidelines from 2017 to 2021. The proportion of cancer survivors who met the aerobic PA guideline of ≥150 min of MVPA per week increased from 35.5% in 2017 to 40.8% in 2020, but it declined to 33.6% in 2021. In contrast, the adherence rate among non-cancer individuals remained relatively stable, averaging 42.3%. Notably, cancer survivors exhibited significantly lower adherence to aerobic activity guidelines compared to non-cancer individuals in 2017 (*p* < 0.01), 2018 (*p* < 0.05), and 2021 (*p* < 0.01).

Regarding adherence to resistance training guidelines (≥2 times per week), cancer survivors consistently showed lower adherence compared to non-cancer individuals. However, in 2017, the meeting attendance rate was slightly higher among cancer survivors (20.4%) compared to non-cancer individuals (19.8%). This pattern was also observed in 2021, with the meeting attendance rate for cancer survivors (24.7%) exceeding that of non-cancer individuals (22.9%). The adherence to combined aerobic and resistance training guidelines was significantly higher among cancer survivors (21.7%) compared to non-cancer individuals (19.3%) in 2019 (Figure 1).

Alongside these results in specific groups, there was also a consistent increase in sedentary behavior among both cancer survivors (from 8.1 h/day in 2017 to 9 h/day in 2021) and non-cancer individuals (from 8.2 h/day in 2017 to 8.9 h/day in 2021; Figure 1).

We also analyzed specific subgroups within the cancer survivor population and found significant differences in the proportion meeting both aerobic and resistance training guidelines. Specifically, survivors who were less than 65 years, male, had higher income and education, lived with a spouse, resided in urban settings, and were within five years of their cancer diagnosis demonstrated a significantly greater likelihood of meeting both aerobic and resistance training guidelines than their counterparts (Supplementary Table S2). Furthermore, given the heterogeneity of cancer types and their potential impact on physical function and overall well-being, we hypothesized that adherence to PA guidelines would vary across cancer types. To investigate this hypothesis, we further stratified by cancer type and found that adherence to aerobic PA guidelines did not differ significantly across cancer types. On the other hand, adherence to resistance training (*p* < 0.01) and to both (aerobic and resistance) PA guidelines (*p* < 0.01) significantly differed across cancer types (Supplementary Table S3).

### 3.3. Patterns of Domain-Specific MVPA Among Cancer Survivors by Sex (2017–2021)

**Figure 2:**
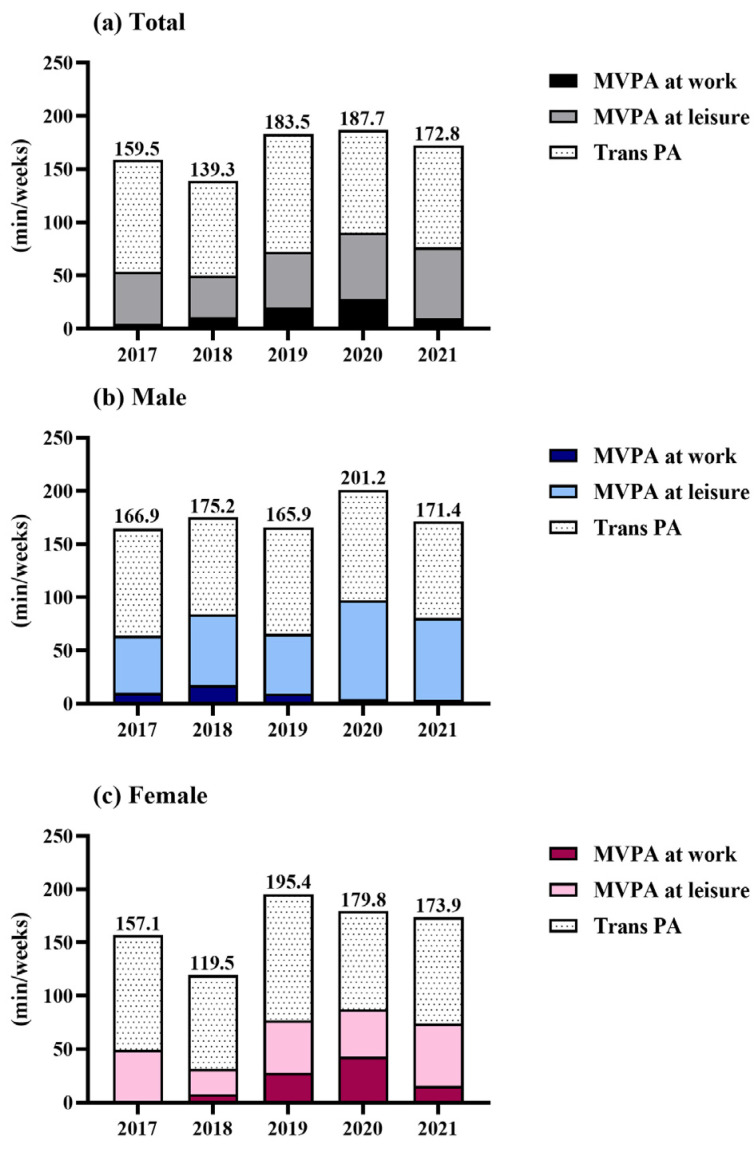
Domain-specific moderate-to-vigorous physical activity (MVPA) among cancer survivors from 2017 to 2021, stratified by sex: (**a**) total cancer survivors, (**b**) male cancer survivors, and (**c**) female cancer survivors. The stacked bar graphs represent the mean weekly minutes of MVPA in different domains: work MVPA, leisure-time MVPA, and transportation (Trans) MVPA. The numbers indicate the total physical activity (PA; sum of all domains) for each year.

Figure 2 presents domain-specific MVPA among cancer survivors, stratified by sex. Analysis of domain-specific PA patterns among cancer survivors revealed that transportation-related PA accounted for the largest proportion of overall activity (mean: 105.2 min/week), followed by leisure-time (mean: 19.8 min/week) and work-related (mean: 4.8 min/week) activities. Furthermore, during the COVID-19 pandemic (2019–2021) significant increases in leisure-time MVPA, total PA, and sedentary time were observed among cancer survivors. Examining trends by sex, men exhibited a decline in work-related MVPA after 2019. In contrast, women experienced a sharp increase in work-related MVPA from 2018 to 2019–2020, followed by a subsequent decline in 2021.

### 3.4. Differences in PA by Treatment Status Among Cancer Survivors

Table 2 presents analyses using medians and interquartile ranges, which revealed that cancer survivors who had completed treatment engaged in significantly more leisure-time MVPA compared to those currently undergoing treatment (*p* = 0.04). Furthermore, significantly higher transportation PA (*p* = 0.01) and total PA (*p* < 0.01) were observed in cancer survivors who had completed treatment compared to those currently undergoing treatment. Domain-specific PA means and standard deviations (SD) are presented in Supplemental Table S1.

### 3.5. Domain-Specific PA and Sedentary Patterns by Time Since Cancer Diagnosis

Analyses stratified by time since cancer diagnosis revealed selective differences across activity domains (Table 3). Significant differences were observed in resistance training frequency (*p* = 0.01), with survivors 3–5 years post-diagnosis showing higher engagement compared to over 6 years post-diagnosis of cancer. However, no significant differences were found in MVPA across work, leisure, and transportation domains or in total PA level between time groups.

Sedentary behavior remained consistently high across all time periods (median: 480 min/day), with no significant differences between groups despite the extended time since diagnosis. Mean and SD values are presented in Supplementary Table S4.

## 4. Discussion

This study examined patterns in PA and sedentary behavior among cancer survivors compared to non-cancer individuals in South Korea from 2017 to 2021. Our key findings were as follows: (1) Cancer survivors consistently demonstrated significantly lower adherence to aerobic PA guidelines than non-cancer individuals in 2017 (*p* < 0.01), 2018 (*p* < 0.05), and 2021 (*p* < 0.01). (2) Transportation-related PA accounted for the majority of the PA among cancer survivors. (3) Sedentary time among cancer survivors also increased, from 8.1 h per day in 2017 to 9 h per day in 2021.

This disparity in PA underscores the challenges cancer survivors face in achieving adequate PA levels, potentially indicating a greater reliance on sedentary behaviors. These challenges may be due to treatment-related side effects [29,30], fatigue [31], or other barriers to exercise adoption and maintenance [8]. Interestingly, the observed decline in aerobic PA adherence among cancer survivors in 2021 warrants further investigation. This decrease coincided with the ongoing COVID-19 pandemic [32], which may have disproportionately affected PA behaviors among this vulnerable population [33]. Restrictions on public spaces [34], concerns about infections [35], and disruptions to regular routines [36] may have contributed to reduced PA levels among cancer survivors [37]. On the other hand, regarding resistance training, the finding that adherence rates among cancer survivors exceeded those of non-cancer individuals in 2021 is noteworthy. This may reflect a shift toward home-based resistance training during the pandemic [13,38,39]. One tentative hypothesis is that, due to concerns about immunity and the increased time spent at home, cancer survivors may have found more opportunities to engage in bodyweight exercises or to use readily available home exercise equipment [13], as reflected in these results. Previous analyses, which indicate that fitness and health care apps gained popularity between 2019 and 2021, support this hypothesis in South Korea [40]. This demonstrated people’s strong preference for the convenience of managing their health through apps, which are easily accessible at home. This trend was further reflected in exercise oncology interventions, which often adapted to a virtually supervised format during the pandemic [41]. Given the known benefits of resistance training for cancer survivors, continued efforts to promote resistance training in this population, particularly accessible and adaptable home-based programs, are warranted.

The analysis of PA domains revealed that transport-related PA constituted the largest proportion of total PA among cancer survivors, followed by leisure-time PA. This pattern suggests that, while cancer survivors may be incorporating some PA into their daily lives through activities such as walking and cycling for transportation, structured exercise programs or dedicated leisure-time activities may be less prevalent. This is significant because structured exercise, in particular, has been shown to yield specific benefits for cancer survivors [31], such as improved cardiorespiratory fitness [42], increased muscle strength [13], and reduced fatigue [31]. Therefore, understanding the reasons for lower engagement in structured exercise, such as access barriers, lack of knowledge, and fear of exacerbating side effects [43], is critical for designing effective and sustainable interventions, which should focus on promoting both active transportation and, importantly, strategies to increase participation in appropriate structured exercise programs that consider the needs and abilities of cancer survivors.

Beyond the observed PA patterns, it is important to note that this pattern may also coincide with increased sedentary time. This could mean that, while survivors are incorporating some activity into their commutes or errands, they may be spending more time sitting at work or home during non-transport hours [44]. This is particularly concerning because prolonged sitting is independently associated with a range of negative health outcomes, including increased risk of cardiovascular disease [45], type-2 diabetes [46], and all-cause mortality [44]. Furthermore, even if cancer survivors meet the recommended PA guidelines, the negative effects of excessive sitting may not be fully mitigated [47]. Actively reducing sedentary time should be a key target of interventions. This can be achieved by incorporating movement breaks into the workday, encouraging active leisure activities, and providing education on the health risks associated with prolonged sitting.

Stratified analyses comparing participants currently receiving cancer treatment to those who had completed treatment revealed lower levels of overall PA among those currently receiving cancer treatment. This disparity underscores the significant challenges individuals undergoing cancer therapy face in maintaining adequate PA levels [48], which likely stem from factors such as cancer-related fatigue [49], lack of clear guidance on safe and effective exercise modalities during specific treatment phases [50], and the resulting lack of awareness among many cancer survivors undergoing active treatment regarding what activities are safe or beneficial [51]. However, a robust body of evidence demonstrates the critical importance of PA during cancer treatment [12]. Numerous studies have shown that exercise interventions during chemotherapy and radiation therapy can lead to significant improvements in physical function [52], cardiorespiratory fitness [53], body composition [15], fatigue [21], mental health [20], and muscle strength [13], thus improving the overall quality of life [54]. Therefore, our findings have important implications for the development and implementation of comprehensive and evidence-based strategies to promote PA among individuals undergoing active cancer treatment.

Our study provides additional insights into the potential barriers to PA adherence among cancer survivors. We observed that cancer survivors who were female, aged 65 or older, more than five years post-diagnosis, had lower income and education levels, or lived without a spouse had lower adherence rates to PA guidelines. These disparities may be attributed to several factors. Studies have shown that older adults often face age-related physical limitations and reduced access to age-appropriate exercise programs [55]. Moreover, women may experience unique barriers because of, for example, caregiving responsibilities [56], safety issues [57], and family duties [57] that limit their leisure-time PA. Lower socioeconomic status is consistently associated with reduced access to affordable exercise facilities that support an active lifestyle [58,59]. Finally, individuals living without a spouse may lack the social support and encouragement needed to maintain regular PA [60,61,62]. Addressing these disparities in PA adherence requires a multi-faceted approach that considers the unique needs and circumstances of these vulnerable subgroups of cancer survivors. The findings of our study, which thoroughly analyzed the PA behaviors of cancer survivors, can provide valuable insights for national public institutions to develop and implement exercise programs that can leverage the established health benefits of PA to improve the outcomes for this vulnerable population.

This study has several limitations that warrant consideration when interpreting the findings. First, while the cross-sectional design provided valuable data on the prevalence of PA and sedentary behavior in the study population, it did not allow us to assess longitudinal changes in these behaviors at the individual level. Second, reliance on the GPAQ introduced potential biases inherent in self-reported data, which may have led to inaccuracies in estimating the actual PA levels. Finally, the analysis was limited by our inability to account for all relevant factors, such as individual diseases. Therefore, caution is warranted when interpreting causal relationships or drawing conclusions about specific subgroups, and future research should consider longitudinal designs and objective measures of PA to validate these findings.

## 5. Conclusions and Clinical Implications

Just as early detection and aggressive treatment are vital during active cancer management, proactive and tailored PA and the reduction of sedentary behavior are essential for optimizing long-term health and well-being. However, this study indicates that cancer survivors consistently demonstrate suboptimal adherence to recommended aerobic exercise guidelines compared to non-cancer individuals.

Given the dominance of transport-related PA and the potential for high levels of overall sedentary time, a comprehensive approach to promoting healthy movement patterns is crucial. Furthermore, our research revealed that survivors who were female, had lower income and education, lived alone, resided in rural settings, and were more than five years from their cancer diagnosis tended to demonstrate lower adherence to PA guidelines. This suggests that survivorship care needs to move beyond simply emphasizing exercise to a comprehensive approach that increases PA and reduces sedentary behavior, addressing this population’s unique needs and circumstances. Moreover, healthcare systems should explore strategies to integrate financial and social support services into survivorship programs, as these factors may indirectly influence PA and sedentary behavior patterns.

Future research should focus on developing and rigorously evaluating culturally appropriate, multi-component interventions that target both individual and environmental factors to foster sustained, meaningful improvements in overall movement behavior, including increased PA and reduced sedentary time, among cancer survivors.

## Figures and Tables

**Table 1:** Demographic and anthropometric profiles of individuals diagnosed with cancer and cancer-free individuals.

	Individuals Diagnosed with Cancer (n = 1585)	Cancer-Free Individuals (n = 26,943)
Arthrometric factor		
Age (years)	63.0 ± 12.5	51.2 ± 17.0
Weight (kg)	61.5 ± 10.8	64.6 ± 13.0
BMI (kg/m^2^)	23.7 ± 3.4	24.0 ± 3.7
WC (cm)	83.7 ± 10.0	83.4 ± 10.6
Male (n, %)	609 (38.4)	11,996 (44.5)
Education level (%)		
≤Middle school	682 (43.0)	7570 (28.1)
High school	487 (30.7)	8999 (33.4)
≥College	416 (26.3)	10,374 (38.5)
Marital status (%)		
Married/live together	1208 (76.2)	17,944 (66.6)
Others *	325 (20.5)	3799 (14.1)
No response	52 (3.3)	248,200 (19.3)
Income (%)		
High	334 (21.1)	3880 (14.4)
Middle-high	319 (20.1)	4823 (17.9)
Middle	325 (20.5)	5577 (20.7)
Middle-low	325 (20.5)	6170 (22.9)
Low	282 (17.8)	6493 (24.1)
Type of cancer (%)		
Gastric	235 (14.8)	-
Colorectal	190 (12.0)	-
Lung	63 (4.0)	-
Thyroid	336 (21.2)	-
Breast	231 (14.6)	-
Liver	33 (2.1)	-
Cervical	125 (7.9)	-
Prostate	74 (4.7)	-
Other	298 (18.8)	-
Currently receiving cancer treatment (%)		
Yes	433 (27.3)	-
Time since cancer diagnosis (%)		
<5 years	574 (36.2)	-

Data are presented as mean ± SD or n (%). * Others = divorced, widowed, and separated. Abbreviations: body mass index, BMI; waist circumference, WC.

**Table 2:** Domain-specific physical activity and sedentary time stratified by current cancer treatment status, gender, and age.

			Currently Receiving Cancer Treatment (n = 433)	Finished Cancer Treatment (n = 1150)	*p*
Total					
		Work MVPA (min/week)	0 (0–0)	0 (0–0)	0.61
		Leisure MVPA (min/week)	0 (0–0)	0 (0–12.50)	0.04
		Transportation PA (min/week)	0 (0–120.0)	20.00 (0–140.00)	0.01
		Total PA (min/week)	40.0 (0–190.0)	90.00 (0–240.00)	0.01
		Resistance training (number/week)	0 (0–0)	0 (0–0)	0.31
		Sedentary behavior (min/week)	480.0 (300.0–660.0)	480.0 (360.0–660.0)	0.28
Male					
		Work MVPA (min/week)	0 (0–0)	0 (0–0)	0.48
		Leisure MVPA (min/week)	0 (0–0)	0 (0–60.0)	0.08
		Transportation PA (min/week)	0 (0–140.0)	0 (0–140.0)	0.60
		Total PA (min/week)	45.0 (0–210.0)	85.0 (0–270.0)	0.31
		Resistance training (number/week)	0 (0–3.0)	0 (0–3.0)	0.43
		Sedentary behavior (min/week)	480.0 (307.5–600.0)	540.0 (360.0–660.0)	0.05
Female					
		Work MVPA (min/week)	0 (0–0)	0 (0–0)	0.91
		Leisure MVPA (min/week)	0 (0–0)	0 (0–0)	0.21
		Transportation PA (min/week)	0 (0–120.0)	40 (0–142.5)	0.01
		Total PA (min/week)	40 (0–180.0)	90.0 (0–210.0)	0.01
		Resistance training (number/week)	0 (0–0)	0 (0–0)	0.60
		Sedentary behavior (min/week)	480.0 (300.0–660.0)	480.0 (300.0–660.0)	0.95
Age					
	(Male) 19–54 years				
		Work MVPA (min/week)	0 (0–0)	0 (0–0)	0.23
		Leisure MVPA (min/week)	0 (0–112.5)	40.0 (0–160.0)	0.30
		Transportation PA (min/week)	0 (0–87.5)	0 (0–210.0)	0.13
		Total PA (min/week)	80.0 (0–175.0)	180.0 (40.0–360.0)	0.05
		Resistance training (number/week)	0.5 (0–3.0)	0 (0–1.0)	0.10
		Sedentary behavior (min/week)	480.0 (330.0–600.0)	600.0 (360.0–720.0)	0.09
	(Male) 55~64 years				
		Work MVPA (min/week)	0 (0–0)	0 (0–0)	0.14
		Leisure MVPA (min/week)	0 (0–17.5)	0 (0–150.0)	0.15
		Transportation PA (min/week)	0 (0–37.5)	0 (0–120.0)	0.27
		Total PA (min/week)	0 (0–255.0)	100.0 (0–360.0)	0.20
		Resistance training (number/week)	0 (0–3.0)	0 (0–3.0)	0.95
		Sedentary behavior (min/week)	450.0 (352.5–720.0)	510.0 (360.0–660.0)	0.61
	(Male) over 65 years				
		Work MVPA (min/week)	0 (0–0)	0 (0–0)	0.31
		Leisure MVPA (min/week)	0 (0–0)	0 (0–0)	0.48
		Transportation PA (min/week)	0 (0–150.0)	0 (0–140.0)	0.64
		Total PA (min/week)	45.0 (0–238.5)	60.0 (0–210.0)	0.76
		Resistance training (number/week)	0 (0–3.0)	0 (0–3.0)	0.72
		Sedentary behavior (min/week)	480.0 (300.0–600.0)	540.0 (360.0–660.0)	0.15
	(Female) 19–54 years				
		Work MVPA (min/week)	0 (0–0)	0 (0–0)	0.51
		Leisure MVPA (min/week)	0 (0–67.5)	0 (0–80.0)	0.44
		Transportation PA (min/week)	0 (0–150.0)	60.0 (0–160.0)	0.16
		Total PA (min/week)	95.0 (0–21.0)	120.0 (0–240.0)	0.25
		Resistance training (number/week)	0 (0–0)	0 (0–0)	0.54
		Sedentary behavior (min/week)	480.0 (300.0–600.0)	480. 0 (300.0–600.0)	0.86
	(Female) 55~64 years				
		Work MVPA (min/week)	0 (0–0)	0 (0–0)	<0.01
		Leisure MVPA (min/week)	0 (0–15.0)	0 (0–60.0)	0.34
		Transportation PA (min/week)	0 (0–150.0)	60.0 (0–162.5)	0.10
		Total PA (min/week)	40.0 (0–205.0)	120.0 (0–300.0)	0.07
		Resistance training (number/week)	0 (0–0)	0 (0–0)	0.68
		Sedentary behavior (min/week)	420.0 (300.0–570.0)	420.0 (300.0–600.0)	0.35
	(Female) over 65 years				
		Work MVPA (min/week)	0 (0–0)	0 (0–0)	0.39
		Leisure MVPA (min/week)	0 (0–0)	0 (0–0)	0.51
		Transportation PA (min/week)	0 (0–92.5)	0 (0–120.0)	0.08
		Total PA (min/week)	0 (0–140.0)	40.0 (0–150.0)	0.05
		Resistance training (number/week)	0 (0–0)	0 (0–0)	0.59
		Sedentary behavior (min/week)	600.0 (390.0–780.0)	540.0 (360.0–720.0)	0.22

Data presented are median (interquartile range (IQR); Q1–Q3); abbreviation: MVPA = moderate to vigorous physical activity.

**Table 3:** Domain-specific physical activity and sedentary time stratified by years since cancer diagnosis and sex.

			≥2 Years (n = 336)	3~5 Years (n = 370)	6~10 Years (n = 463)	≤11 Years (n = 416)	*p*
Total							
		Work MVPA (min/week)	0 (0–0)	0 (0–0)	0 (0–0)	0 (0–0)	0.39
		Leisure MVPA (min/week)	0 (0–0)	0 (0–30.0)	0 (0–30.0)	0 (0–0)	0.07
		Transportation PA (min/week)	0 (0–120.0)	0 (0–150.0)	0 (0–120.0)	30.0 (0–150.0)	0.39
		Total PA (min/week)	60.0 (0–207.5)	80.0 (0–215.0)	70.0 (0–210.0)	80.0 (0–240.0)	0.93
		Resistance training (number/week)	0 (0–0)	0 (0–2.0)	0 (0–0) *	0 (0–0) *	0.01
		Sedentary behavior (min/week)	480.0 (360.0–660.0)	480.0 (300.0–600.0)	480.0 (360.0–660.0)	480.0 (330.0–660.0)	0.66
Male							
		Work MVPA (min/week)	0 (0–0)	0 (0–0)	0 (0–0)	0 (0–0)	0.45
		Leisure MVPA (min/week)	0 (0–85.0)	0 (0–32.5)	0 (0–62.5)	0 (0–0)	0.65
		Transportation PA (min/week)	0 (0–130.0)	0 (0–120.0)	0 (0–120.0)	40.0 (0–170.0)	0.08
		Total PA (min/week)	60.0 (0–245.0)	60.0 (0–217.5)	65.0 (0–252.5)	90.0 (0–290.0)	0.88
		Resistance training (number/week)	0 (0–0)	0 (0–3.0)	0 (0–1.0)	0 (0–3.0)	0.97
		Sedentary behavior (min/week)	480.0 (360.0–660.0)	525.0 (360.0–637.5)	540.0 (360.0–720.0)	510.0 (360.0–660.0)	0.95
Female							
		Work MVPA (min/week)	0 (0–0)	0 (0–0)	0 (0–0)	0 (0–0)	0.74
		Leisure MVPA (min/week)	0 (0–0)	0 (0–30.0)	0 (0–0)	0 (0–0)	0.14
		Transportation PA (min/week)	20.0 (0–120.0)	40.0 (0–175.0)	20.0 (0–120.0)	30.0 (0–140.0)	0.56
		Total PA (min/week)	60.0 (0–180.0)	90.0 (0–220.0)	70.0 (0–210.0)	75.0 (0–210.0)	0.85
		Resistance training (number/week)	0 (0–0)	0 (0–0)	0 (0–0)	0 (0–0)	0.17
		Sedentary behavior (min/week)	495.0 (352.5–697.5)	420.0 (300.0–600.0)	480.0 (330.0–660.0)	480.0 (310.0–660.0)	0.16

Data presented are median (interquartile range (IQR); Q1–Q3), * = significantly different from 3–5 years after cancer diagnosis (*p* < 0.05, Bonferroni post hoc test). Abbreviations: MVPA = moderate to vigorous physical activity, PA = physical activity.

## Data Availability

The original contributions presented in this study are included in the article. Further inquiries can be directed to the corresponding author.

## Supplementary Materials

The following supporting information can be downloaded at: https://www.mdpi.com/article/10.3390/cancers17142270/s1, Figure S1: Flow diagram; Table S1: Domain-specific physical activity and sedentary time stratified by current cancer treatment status, gender, and age; Table S2: Physical activity guideline adherence by subgroup among individuals diagnosed with cancer; Table S3: Adherence to physical activity guidelines, domain-specific activity levels, and sedentary behavior by cancer type; Table S4: Domain-specific physical activity and sedentary time stratified by years since cancer diagnosis, sex, and age.

## Abbreviations

The following abbreviations are used in this manuscript:

BMI	Body-mass index
GPAQ	Global Physical Activity Questionnaire
KNHANES	Korea National Health and Nutrition Examination Survey
MVPA	Moderate-to-vigorous physical activity
PA	Physical activity
SD	Standard deviation
WC	Waist circumference
