# Supplementary material for: Physical Activity and Sedentary Behavior Patterns Among Korean Cancer Survivors: A Cross-Sectional Analysis (2017–2021)
Source: Cancers (Basel). 2025 Jul 8;17(14):2270. doi: 10.3390/cancers17142270 (PMC12293402; doi:10.3390/cancers17142270)
Supplement: Supplementary file 1 [file cancers-17-02270-s001.zip › cancers-3696892-supplementary.pdf]

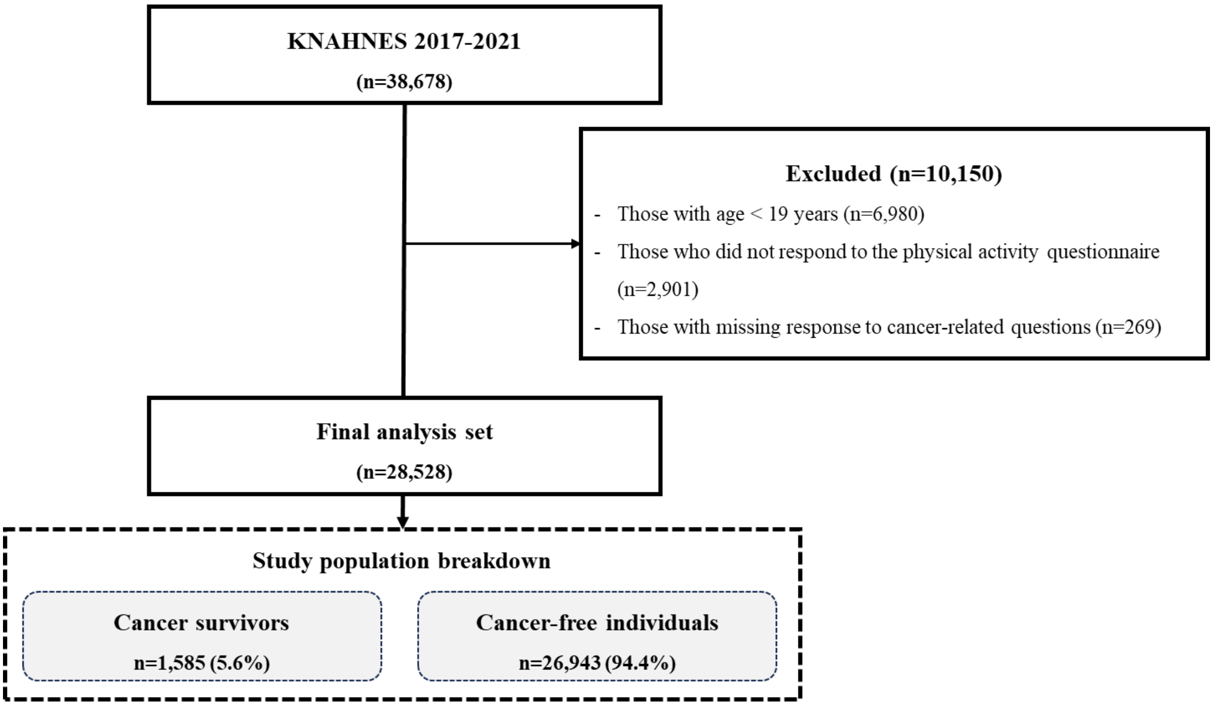

Figure S1. Flow diagram

Table S1. Domain-specific physical activity and sedentary time stratified by current cancer treatment status, gender, and age

	Current receiving cancer treatment (n=433)	Current finished cancer treatment (n=1,150)	*P*
Total			
MVPA at work (min/week)	13.2±90.5	16.3±159.5	0.61
MVPA at leisure (min/week)	46.7±116.1	57.8±143.5	0.04
Transportation PA (min/week)	96.5±202.6	100.9±184.3	0.01
Total PA (min/week)	153.4±266.6	174.9±304.0	<0.01
Resistance training (number/week)	0.9±1.7	0.8±1.6	0.31
Sedentary behavior (min/week)	498.0±218.3	511.2±223.2	0.28
Male			
MVPA at work (min/week)	6.3±45.3	10.4±73.8	0.48
MVPA at leisure (min/week)	57.3±143.4	74.2±171.5	0.08
Transportation PA (min/week)	101.3±209.2	95.4±174.7	0.60
Total PA (min/week)	164.8±262.0	179.8±269.8	0.31
Resistance training (number/week)	1.4±2.0	1.2±1.9	0.43
Sedentary behavior (min/week)	495.4±215.9	531.8±220.2	0.05
Female			
MVPA at work (min/week)	17.82±110.5	19.9±1943.0	0.91
MVPA at leisure (min/week)	34.7±93.1	47.8±122.5	0.21
Transportation PA (min/week)	93.3±198.5	104.2±190.0	<0.01
Total PA (min/week)	145.8±269.8	171.9±323.3	<0.01
Resistance training (number/week)	0.6±1.4	0.6±1.4	0.60
Sedentary behavior (min/week)	499.7±220.3	498.6±224.2	0.95
Age			
(Male) 19~54 years			
MVPA at work (min/week)	0	24.7±129.9	0.23
MVPA at leisure (min/week)	70.8±124.9	94.9±126.2	0.30
Transportation PA (min/week)	51.7±87.5	102.6±134.8	0.13
Total PA (min/week)	122.5±151.6	222.2±223.6	0.05
Resistance training (number/week)	1.5±1.9	0.8±1.5	0.10
Sedentary behavior (min/week)	466.3±173.4	553.7±203.3	0.09
(Male) 55~64 years			
MVPA at work (min/week)	0	20.6±103.9	0.14
MVPA at leisure (min/week)	65.6±148.9	112.6±246.4	0.15
Transportation PA (min/week)	80.9±187.1	97.5±213.8	0.27
Total PA (min/week)	146.5±217.6	230.7±360.3	0.20

Resistance training (number/week)	1.3±1.9	1.3±1.9	0.95
Sedentary behavior (min/week)	506.5±242.1	518.4±218.8	0.61
(Male) over 65 years			
MVPA at work (min/week)	9.5±55.5	3.6±33.5	0.31
MVPA at leisure (min/week)	51.9±146.2	54.8±135.7	0.48
Transportation PA (min/week)	117.8±231.3	93.3±163.9	0.64
Total PA (min/week)	179.2±291.0	151.3±228.7	0.76
Resistance training (number/week)	1.4±2.1	1.2±2.0	0.72
Sedentary behavior (min/week)	498.4±217.0	533.3±224.2	0.15
(Female) 19~54 years			
MVPA at work (min/week)	29.3±149.4	29.8±239.8	0.51
MVPA at leisure (min/week)	51.8±97.9	64.3±133.5	0.44
Transportation PA (min/week)	99.0±180.4	111.0±200.2	0.16
Total PA (min/week)	180.0±271.4	205.1±339.3	0.25
Resistance training (number/week)	0.6±1.3	0.5±1.2	0.54
Sedentary behavior (min/week)	467.0±207.6	470.4±216.6	0.86
(Female) 55~64 years			
MVPA at work (min/week)	20.7±121.6	0	<0.01
MVPA at leisure (min/week)	45.5±111.3	70.8±151.8	0.34
Transportation PA (min/week)	124.2±278.2	114.7±180.1	0.10
Total PA (min/week)	190.3±360.1	185.5±238.9	0.07
Resistance training (number/week)	0.6±1.4	0.8±1.6	0.68
Sedentary behavior (min/week)	429.2±187.3	454.6±196.2	0.35
(Female) over 65 years			
MVPA at work (min/week)	6.5±46.2	26.9±223.5	0.39
MVPA at leisure (min/week)	12.9±67.2	19.6±77.4	0.51
Transportation PA (min/week)	65.4±124.7	91.9±189.1	0.08
Total PA (min/week)	84.8±156.7	138.5±359.5	0.05
Resistance training (number/week)	0.6±1.5	0.4±1.2	0.59
Sedentary behavior (min/week)	584.7±229.6	552.9±238.7	0.22

Data are present Mean±SD, *P*= Mann-Whitney U tests, abbreviation: PA=physical activity, MVPA=moderate to vigorous physical activity

Table S2. Physical activity guideline adherence by subgroup among individuals diagnosed with cancer

Subgroup			*P*
Age	≤65 years old	>65 years old	
Meeting for aerobic PA guideline	44.1	29.9	<.001
Meeting for resistance training guideline	20.4	20.2	0.95
Meeting for both PA guideline	11.5	8.9	<.001
Gender	Male	Female	
Meeting for aerobic PA guideline	38.6	36.1	0.34
Meeting for resistance training guideline	29.3	14.7	<0.01
Meeting for both PA guideline	14.4	7.6	<0.01
Obesity	Normal weight	Over-weight & Obese	
Meeting for aerobic PA guideline	39.6	34.8	0.06
Meeting for resistance training guideline	21.4	19.6	0.41
Meeting for both PA guideline	11.5	9.0	0.22
Income	Low	High	
Meeting for aerobic PA guideline	31.3	43.9	<0.01
Meeting for resistance training guideline	18.4	22.6	0.04
Meeting for both PA guideline	8.3	12.5	<0.01
Education level	≤High school	>college	
Meeting for aerobic PA guideline	33.5	47.5	<0.01
Meeting for resistance training guideline	18.8	24.6	0.02
Meeting for both PA guideline	8.7	14.5	<0.01
Material status	Live with spouse	Live alone	
Meeting for aerobic PA guideline	39.3	28.5	<0.01
Meeting for resistance training guideline	22.0	13.4	<0.01
Meeting for both PA guideline	11.0	6.3	<0.01
Residence type	City	Rural	

Meeting for aerobic PA guideline	42.0	33.4	<0.01
Meeting for resistance training guideline	22.1	19.0	0.15
Meeting for both PA guideline	12.0	8.87	<.01
Current treatment	Yes	No (finish)	
Meeting for aerobic PA guideline	38.2	34.0	0.13
Meeting for resistance training guideline	19.8	21.8	0.4
Meeting for both PA guideline	10.4	9.7	0.27
Time since diagnosis cancer	≤5years	> 5years	
Meeting for aerobic PA guideline	37.7	36.7	0.71
Meeting for resistance training guideline	24.5	17.8	<0.01
Meeting for both PA guideline	12.1	9.1	0.02

Data are present %, Obesity was classified based on BMI ≥ 23 kg/m^2, with individuals categorized as either normal weight (BMI < 23 kg/m^2) or overweight/obese (BMI ≥ 23 kg/m^2), according to the Asia-Pacific BMI classification. Income level was categorized based on quintiles. Participants in the low, lower-middle, and middle quintiles were grouped as "low" income, while those in the upper-middle and high quintiles were classified as "high" income

Table S3. Adherence to physical activity guidelines, domain-specific activity levels, and sedentary behavior by cancer type

	Gastric cancer (n=235)	Colorectal cancer (n=190)	Lung cancer (n=63)	Thyroid cancer (n=336)	Breast cancer (n=231)	Liver Cancer (n=33)	Cervical cancer (n=125)	Prostate cancer (n=)	etc. (n=298)	*P*
Adherence to PA guidelines (%)										
Meeting for aerobic PA guideline	30.8	39.0	38.1	41.4	38.4	37.5	31.0	38.7	36.5	0.33
Meeting for resistance training guideline	20.0	27.9	20.6	16.4	12.9	40.6	11.1	26.7	25.7	<0.01
Meeting for both PA guideline	8.9	12.1	15.9	8.3	7.3	21.9	4.8	13.3	13.2	<0.01

Data are present % or median (Interquartile range (IQR); Q1-Q3), etc=encompasses malignancies excluding gastric, colorectal, lung, thyroid, breast, liver, cervical, and prostate cancers (e.g., ovarian, renal, bladder, esophageal, and pancreatic cancers). abbreviation: PA=physical activity, MVPA=moderate to vigorous physical activity

Table S4. Domain-specific physical activity and sedentary time stratified by years since cancer diagnosis, sex, and age

	≥ 2 years (=336)	3~5 years (n=370)	6~10 years (n=463)	≤ 11 years (n=416)	*P*
Total					
MVPA at work (min/week)	11.0±79.6	14.8±91.0	17.9±189.3	16.6±161.9	0.39
MVPA at leisure (min/week)	60.7±137.8	54.5±130.3	57.8±141.6	43.0±134.7	0.07
Transportation PA (min/week)	80.5±139.2	106.3±218.5	91.6±167.0	118.7±218.4	0.39
Total PA (min/week)	152.3±224.9	175.3±275.4	167.2±3232.2	178.3±324.6	0.93
Resistance training (number/week)	0.9±1.8	1.0±1.8	0.7±1.5*	0.7±1.6*	0.01
Sedentary behavior (min/week)	518.4±224.1	496.0±224.5	513.1±218.5	503.6±222.7	0.66
Male					
MVPA at work (min/week)	6.5±43.9	19.6±110.7	6.9±45.6	1.4±13.7	0.45
MVPA at leisure (min/week)	78.2±163.7	56.1±133.2	76.1±163.5	63.6±197.8	0.65
Transportation PA (min/week)	83.7±159.3	98.9±210.7	84.1±139.8	133.1±234.2	0.08
Total PA (min/week)	168.4±241.3	174.0±272.3	167.1±242.1	198.1±325.2	0.88
Resistance training (number/week)	1.2±2.0	1.5±2.1	1.0±1.7	1.2±2.0	0.97
Sedentary behavior (min/week)	513.0±222.7	518.6±217.6	539.5±215.7	512.9±226.2	0.95
Female					
MVPA at work (min/week)	14.9±100.0	10.9±70.9	24.5±236.7	22.7±191.3	0.74
MVPA at leisure (min/week)	46.2±110.1	53.1±128.3	46.8±125.5	34.8±97.8	0.14
Transportation PA (min/week)	77.9±120.2	112.3±224.9	96.0±181.6	112.9±211.9	0.56
Total PA (min/week)	138.9±210.1	176.3±278.5	167.3±363.8	170.3±324.5	0.85
Resistance training (number/week)	0.6±1.5	0.7±1.4	0.5±1.3	0.5±1.3	0.17
Sedentary behavior (min/week)	522.9±225.8	477.5±228.8	497.2±219.1	499.8±221.5	0.16

Data are present Mean±SD, *=significantly different from 3-5 years after cancer diagnosis ($p<0.05$, Bonferroni post hoc test), abbreviation: PA=physical activity, MVPA=moderate to vigorous physical activity
